# Integrated Microarray and RNAseq Transcriptomic Analysis of Retinal Pigment Epithelium/Choroid in Age-Related Macular Degeneration

**DOI:** 10.3389/fcell.2020.00808

**Published:** 2020-08-21

**Authors:** Dhanach Dhirachaikulpanich, Xin Li, Louise F. Porter, Luminita Paraoan

**Affiliations:** ^1^Department of Eye and Vision Science, Institute of Life Course and Medical Sciences, University of Liverpool, Liverpool, United Kingdom; ^2^Faculty of Medicine, Siriraj Hospital, Mahidol University, Bangkok, Thailand

**Keywords:** age-related macular degeneration, retinal pigment epithelium, neurodegeneration, transcriptome, neuroactive ligand-receptor, extracellular matrix

## Abstract

We report for the first time an integrated transcriptomic analysis of RPE/choroid dysfunction in AMD (mixed stages) based on combining data from publicly available microarray (GSE29801) and RNAseq (GSE135092) datasets aimed at increasing the ability and power of detection of differentially expressed genes and AMD-associated pathways. The analysis approach employed an integrating quantitative method designed to eliminate bias among different transcriptomic studies. The analysis highlighted 764 meta-genes (366 downregulated and 398 upregulated) in macular AMD RPE/choroid and 445 meta-genes (244 downregulated and 201 upregulated) in non-macular AMD RPE/choroid. Of these, 731 genes were newly detected as differentially expressed (DE) genes in macular AMD RPE/choroid and 434 genes in non-macular AMD RPE/choroid compared with controls. Over-representation analysis of KEGG pathways associated with these DE genes mapped revealed two most significantly associated biological processes in macular RPE/choroid in AMD, namely the neuroactive ligand-receptor interaction pathway (represented by 30 DE genes) and the extracellular matrix-receptor interaction signaling pathway (represented by 12 DE genes). Furthermore, protein-protein interaction (PPI) network identified two central hub genes involved in the control of cell proliferation/differentiation processes, *HDAC1* and *CDK1*. Overall, the analysis provided novel insights for broadening the exploration of AMD pathogenesis by extending the number of molecular determinants and functional pathways that underpin AMD-associated RPE/choroid dysfunction.

## Introduction

The pathogenesis of age-related macular degeneration (AMD), a leading cause of irreversible blindness in the world, is linked to degenerative changes in the retina, retinal pigment epithelium (RPE) and choroid. Major risk factors for AMD are advanced age, family history and smoking (Klein et al., 2007; Wang et al., 2007). At the cellular level, DNA damage, oxidative stress, inflammation, mitochondrial dysfunction, cellular senescence, abnormal metabolism, and aberrant proteolysis contribute to AMD development (Kay et al., 2014; Wang et al., 2019; Blasiak, 2020). Located between the neuroretina and choriocapillaris, the RPE is a major tissue involved in pathogenesis sustaining retinal function through metabolite exchanges, protein secretion, phagocytosis of spent photoreceptor outer segments, and immune barrier function through interaction with Bruch’s membrane, the basement membrane of the RPE (Strauss, 2005; Sparrow et al., 2010). Impaired RPE function has been shown to precede photoreceptors’ death in AMD, leading to progressive degeneration of the neuroretina. Accumulation of medium and large-size drusen, lipo-proteinaceous deposits found below the RPE’s basement membrane (Mitchell et al., 2018; Wang et al., 2019; Blasiak, 2020) is a significant factor in AMD progression from early to the advance disease, evidenced by population-based cohorts (Klein et al., 2007; Wang et al., 2007). The choriocapillaris, a vascular endothelium situated just beneath the RPE and Bruch’s membrane provides nutrients and oxygenation to the RPE (Whitmore et al., 2015) and also represents a major site of age-related degenerative changes with reduced vascular endothelial density (Ramrattan et al., 1994), vulnerability to inflammation through the membrane attack complex with increasing age, together contributing to AMD (Mullins et al., 2014). However, to date the precise molecular mechanisms of AMD pathogenesis and progression from early to advanced stages are incompletely understood (Ardeljan and Chan, 2013). Significant amount of research in recent years has concentrated on the complement pathway and inflammatory processes, but new emerging treatments targeting only the complement pathway failed to improve clinical outcomes in phase 3 trials (Mitchell et al., 2018). Clearly, an integrated research approach considering other contributing pathogenic mechanisms is needed to identify novel and viable therapeutic targets.

Transcriptomic data, gathered by microarray (Booij et al., 2009; Newman et al., 2012; Whitmore et al., 2013), RNAseq (Whitmore et al., 2014; Kim et al., 2018) or very recently advanced single-cell (sc)RNAseq (Voigt et al., 2019; Orozco et al., 2020) studies provide a solid starting point for the study of the molecular determinants of RPE/choroid dysfunction in AMD (Morgan and DeAngelis, 2014; Tian et al., 2015). Publicly available transcriptomic datasets allow targeted analyses of specific cellular processes, pathways, and their interactions. To date, transcriptomic RPE/choroid analyses focused on topographic regions, specifically macular versus non-macular retinal regions, have revealed different transcription profiles in these regions associated with various macular dystrophies and degenerative retinal diseases, including Best disease, Stargardt’s disease and retinitis pigmentosa (Whitmore et al., 2014; Ashikawa et al., 2017). However, identification of the causative differentially expressed genes between AMD and age-matched controls from individual experiments is far from conclusive to date, conceivably due to the relatively small sample sizes of many datasets often compounded by AMD phenotype heterogeneity within the datasets [early and advanced AMD, geographic atrophy (GA), and neovascular (NV) AMD samples] and further confounded by the transcriptomic characteristics of aging biology (De Magalhães et al., 2009; Whitmore et al., 2013; Orozco et al., 2020). This is reflected in the generally small overlap between differentially expressed genes from specific AMD datasets. Other confounding factors may also include different sample preparation methods, transcriptomic platforms and data analysis methods employed across different studies (Tian et al., 2015).

An integrating quantitative method of analysis of combined datasets can eliminate bias between transcriptomic studies and increase the power of detection of differentially expressed genes (Zhou et al., 2016; Brown et al., 2017; Ma et al., 2017; Alimadadi et al., 2020). Here, we describe such an analysis approach applied to investigate different platforms of publicly available transcriptomic datasets of post-mortem human AMD RPE/choroid. The differential gene expression patterns, pathway analysis and networks of protein-protein interactions (PPI) were explored in the combined datasets.

## Materials and Methods

### Data Collection

Publicly available post-mortem human AMD RPE/choroid transcriptome datasets were accessed through the NCBI GEO and ArrayExpress databases combined with a literature review for individual datasets. The post-mortem human AMD RPE/choroid transcriptome data generated by microarrays and RNAseq were selected and filtered using the following criteria: (1) data published between January 2010 and February 2020; (2) complete gene expression data available (raw or normalized); (3) sample size equal or higher than 10 in each group (AMD and control); (4) original specimens divided into macular and non-macular samples. Only two datasets passed these criteria and were included in our study, GSE135092 and GSE29801. GSE135092 originated from an RNAseq study performed by Illumina HiSeq2500. The respective gene expression data provided by this dataset was quantified by HTSeqGenie as reads per kilobase of gene model per million total reads (RPKM), then normalized by DESeq2 (Orozco et al., 2020). GSE29801 dataset originated from a study using the Agilent G4112F array, obtained after quality control, background subtraction, and normalization as described by Newman et al. (2012).

### Data Analysis

**FIGURE 1:**
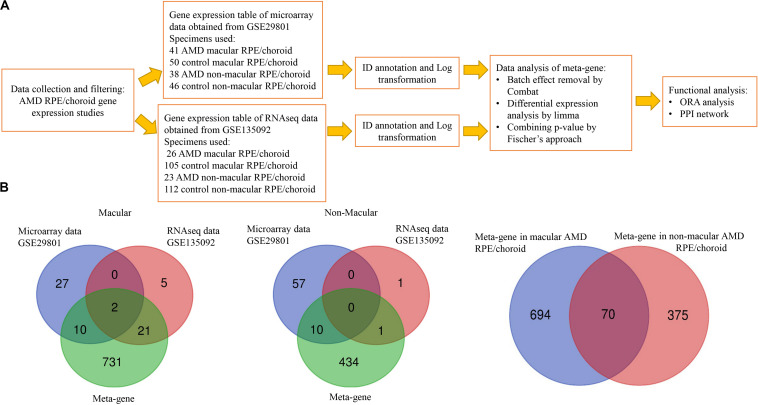
Data processing workflow and common genes between the datasets analyzed. **(A)** Data processing workflow. **(B)** Venn diagram showing the overlap of differentially expressed genes identified by microarrays, RNAseq and meta-gene in macular and non-macular locations of RPE/choroid in AMD.

To integrate the different study platforms, we used the two-step conventional metanalysis approach described by Ma et al. (2017) For each platform, individual analyses were performed separately using the appropriate and specific bioinformatics pipeline for the respective application (e.g., edgeR or DESeq2 or limma for RNAseq and limma for microarray). We then combined the *p*-values obtained, setting the statistical significance threshold for each gene based on the result of this combined *p*-value (Tseng et al., 2012). The combined *p*-value is widely used in meta-analysis statistics of differential expressed genes since it is simple and versatile – it was shown to be applicable to analysis of both multiple microarray datasets and combined microarray and RNAseq datasets (Tseng et al., 2012; Ma et al., 2017). The diagram of data processing is shown in Figure 1. The gene expression table from each individual dataset was annotated and analyzed by the web-based analysis tool Networkanalyst^1^ (Xia et al., 2014, 2015; Zhou et al., 2019). The identifiers (IDs) from different platforms (ENSEMBL gene IDs for RNAseq and probe IDs for microarrays) were converted to Entrez gene IDs. The log transformation by variance stabilizing normalization (VSN) in combination with quantile normalization was performed for microarray data. Similarly, RNAseq data were transformed to log2 counts per million by the log2 count procedure. Differential expression (DE) analysis of each study was performed by limma using adjusted *p* < 0.05 from Benjamini-Hochberg’s False Discovery Rate (FDR) (Ritchie et al., 2015). To make data comparable, the batch effect between studies was minimized using the ComBat algorithm and then examined by principal component analysis (PCA) (Supplementary Figures S1, S2; Johnson et al., 2007). The batch effect removal algorithm (ComBat) was also beneficial in background noise reduction, through the removal of genes with totally absent expression in more than 80 percent of samples whilst equally reducing the variability of gene expression levels between batches (Johnson et al., 2007; Zhou et al., 2016). Using Fischer’s approach for meta-analysis, each study *p*-value was combined together using the formula below.

$$F_{g}=-2\,\sum_{s=1}^{s}\left(\ln\left(P_{g\,s}\right)\right)$$

A calculated combined *p*-value for each gene was considered significant if lower than 0.05 (Fisher, 1992; Xia et al., 2015; Alimadadi et al., 2020). The differential significant gene list obtained was then called the meta-gene dataset in this paper.

### Data Interpretation and Functional Analysis

The resulting meta-gene list was compared with the original DE gene list in each of the original studies. To identify significant pathways from the meta-gene list, over-representation analysis (ORA) was performed using Kyoto Encyclopedia of Genes and Genomes (KEGG) and *p*-values were adjusted by Benjamini-Hochberg’s False Discovery Rate (FDR). A protein-protein interaction (PPI) network was constructed based on STRING database (Szklarczyk et al., 2019) and then visualized by a web-based tool^1^ (Xia et al., 2014, 2015; Zhou et al., 2019). Hub nodes were identified by high degrees and high centrality from the PPI network. The results were then compared with the network constructed by WEB-based GEne SeT AnaLysis Toolkit (Zhang et al., 2005; Wang et al., 2013, 2017; Liao et al., 2019).

## Results

### RPE/Choroid AMD Transcriptomic Datasets

Five post-mortem human RPE/choroid AMD transcriptome studies were identified through the literature review shown in Supplementary Table S1: two microarrays studies, one RNAseq, one scRNAseq and a recent study using both RNAseq and scRNAseq. All of these datasets were accessible through the NCBI GEO database but only two fulfilled our inclusion criteria, as follows. The dataset GSE29801 was generated by a study using Agilent Whole Human Genome 4 × 44K *in situ* oligonucleotide array platform (G4112F array) (Newman et al., 2012). In this study eyes with either a clinical or pathological diagnosis of AMD and with age ranging from 43 to 101 years were analyzed making use of 41 AMD macular RPE/choroid specimens [9 advanced AMD, 16 intermediate, 10 early and 6 undefined stage using the Age Related Eye Diseases (AREDS) classification], 50 control macular RPE/choroid specimens, 38 AMD non-macular RPE/choroid specimens (9 advanced, 14 intermediate, 9 early and 6 undefined stage) and 46 control non-macular RPE/choroid specimens. The GSE135092 dataset was provided by an RNAseq study of eyes with a clinical diagnosis of AMD using the AREDS classification and ages ranging from 59 to 98 years, performed using the Illumina HiSeq2500 platform (Orozco et al., 2020). The study analyzed 26 AMD macular RPE/choroid specimens (mixed advanced stages), 105 control macular RPE/choroid specimens, 23 AMD non-macular RPE/choroid specimens and 112 control non-macular RPE/choroid specimens (Figure 1).

### Meta-Gene Dataset

The DE genes identified as a result of the combined analysis are referred to as meta-genes. DE genes were analyzed by integration of the two selected datasets through Networkanalyst web-based software. Initial analysis of individual datasets by limma with an FDR < 0.05 found only 10 DE genes (Supplementary Table S2) in macular and 57 DE genes in non-macular AMD RPE/choroid (Supplementary Table S3). To further interrogate the differences between AMD and control RPE/choroid, a more sensitive method involving Fischer’s approach was then applied to the integrated data. After data normalization and batch effect adjustment, the PCA plot did not show major differences between studies, which indicated that the batch effect was reduced between the two studies (Supplementary Figures S1, S2). By using Fisher’s approach for combining *p*-value, 764 significant meta-genes (366 down-regulated and 398 up-regulated) were detected in macular AMD RPE/choroid (Supplementary Table S4) and 445 meta-genes (244 down-regulated and 201 up-regulated) in non-macular AMD RPE/choroid (Supplementary Table S5). By ranking the combined *p*-values, the top 20 significant genes in macular and non-macular AMD RPE/choroid, respectively, were obtained and shown in Table 1.

**TABLE 1:** Meta-gene list showing top differentially expressed genes.

EntrezID	Gene symbol	F_g_	Combined *p*-value	Effect
Differential expressed genes identified in macular AMD RPE/choroid vs. macular control RPE/choroid				
84624	FNDC1	–47.991	1.72E -05	Up-regulated
4060	LUM	–46.502	1.75E -05	Up-regulated
131578	LRRC15	–40.042	0.00019	Up-regulated
5803	PTPRZ1	–38.086	0.00032	Up-regulated
9547	CXCL14	–38.22	0.00032	Up-regulated
8148	TAF15	–35.072	0.00102	Up-regulated
4804	NGFR	–34.669	0.00109	Up-regulated
3381	IBSP	–31.756	0.00272	Up-regulated
3371	TNC	–31.912	0.00272	Up-regulated
1118	CHIT1	–31.852	0.00272	Up-regulated
1515	CTSV	–31.612	0.00272	Up-regulated
84466	MEGF10	–31.106	0.00278	Up-regulated
2224	FDPS	–31.247	0.00278	Up-regulated
6695	SPOCK1	–30.827	0.00287	Up-regulated
55827	DCAF6	40.05	0.00019	Down-regulated
64093	SMOC1	37.387	0.00039	Down-regulated
7066	THPO	31.517	0.00272	Down-regulated
100128731	OST4	31.747	0.00272	Down-regulated
2619	GAS1	32.158	0.00272	Down-regulated
83473	KATNAL2	31.124	0.00278	Down-regulated

**Differential expressed genes identified in non-macular AMD RPE/choroid vs. non-macular control RPE/choroid**				

54108	CHRAC1	–40.355	0.00066	Up-regulated
10648	SCGB1D1	–36.412	0.00216	Up-regulated
64116	SLC39A8	–31.469	0.00826	Up-regulated
84656	GLYR1	–30.629	0.00826	Up-regulated
79095	C9orf16	–30.731	0.00826	Up-regulated
6422	SFRP1	–28.224	0.01414	Up-regulated
1974	EIF4A2	32.756	0.0081	Down-regulated
58155	PTBP2	30.667	0.00826	Down-regulated
400073	C12orf76	31.225	0.00826	Down-regulated
146225	CMTM2	29.187	0.0118	Down-regulated
65982	ZSCAN18	29.314	0.0118	Down-regulated
23564	DDAH2	29.244	0.0118	Down-regulated
115761	ARL11	28.924	0.01223	Down-regulated
6404	SELPLG	27.816	0.01414	Down-regulated
84695	LOXL3	27.903	0.01414	Down-regulated
8936	WASF1	27.635	0.01414	Down-regulated
8675	STX16	27.52	0.01414	Down-regulated
8803	SUCLA2	27.535	0.01414	Down-regulated
54816	ZNF280D	28.257	0.01414	Down-regulated
3187	HNRNPH1	27.798	0.01414	Down-regulated

The extent of overlap between meta-genes and original DE genes detected in each study (Supplementary Tables S2, S3) is shown in the Venn diagrams in Figure 1. A higher degree of overlap was identified in macular AMD RPE/choroid, with *PRSS33* and *SMOC1* detected as common DE genes in all datasets. No overlap of DE genes was detected between all three groups of genes in non-macular AMD RPE/choroid. Thirty-one genes were common between the microarray or RNAseq datasets, and the meta-genes of macular AMD RPE/choroid, while 11 common genes were detected in non-macular AMD RPE/choroid. In our analysis, 731 genes were newly detected as DE genes in macular and 434 genes in non-macular AMD RPE/choroid. Among the meta-genes, 70 genes were similarly differentially expressed in both macular and non-macular AMD RPE/choroid (Supplementary Table S6).

Furthermore, because AMD samples in GSE135092 consisted of mixed advanced stages of AMD (GA and NV AMD), and samples in GSE29801 consisted of advanced stages (GA and NV AMD), intermediate, and early stage of AMD, we performed subgroup analysis combining each AMD stage subgroup (early, intermediate, “mixed” advanced AMD) from GSE29801 with all GSE135092 samples (Supplementary Table S7). Interestingly, the presence of advanced AMD predominantly influenced the expression of genes included in the 764 meta-genes identified as DE in macular RPE/choroid, a stepwise reducing trend identified in intermediate then early stage of AMD, respectively (Supplementary Figure S3). However, to maximize the number of samples and therefore power in this analysis, we used the meta-genes from all RPE/choroid samples in further downstream analyses.

### KEGG Pathway Analysis

To interrogate the functional significance of meta-genes, over-representation analyses (ORA) of KEGG pathways were applied to both macular and non-macular meta-genes identified. Applying FDR < 0.05, the interactions with the neuroactive ligand-receptor and the extracellular matrix (ECM)-receptor interaction pathways were statistically significant in macular AMD RPE/choroid, while there was no statistically significant pathway identified in non-macular AMD RPE/choroid. Table 2 shows the top 5 KEGG pathways and meta-genes in each pathway found in macular and non-macular AMD RPE/choroid.

**TABLE 2:** ORA analysis showing top KEGG pathways involving the meta-genes.

Pathway	*p*-value	FDR	Differential expressed gene (gene symbol)
**Macular AMD RPE/choroid vs. macular control RPE/choroid**			
Neuroactive ligand-receptor interaction	0.000126	0.0297	CHRNA1; GRIA1; OXTR; GABRB1; NPFFR1; SCT; GRIK3; ADRA1D; TRH; HTR2A; GRPR; ADRA1A; C5; P2RY2; PENK; LEPR; BDKRB2; BDKRB1; GABRE; PTGDR; CHRNB4; EDN3; GCGR; NPY1R; GRIN2C; GABRG3; MTNR1A; ADRB3; MC5R;RLN3
ECM-receptor interaction	0.000187	0.0297	COMP; RELN; IBSP; ITGB4; ITGA3; TNC; SPP1; COL6A3; COL9A3; COL9A2; THBS2; THBS4
AMPK signaling pathway	0.000626	0.0664	SREBF1; CAB39L; IRS2; PPP2R3A; FOXO3; EEF2; ADRA1A; G6PC2; PFKL; SCD; FASN; LEPR; PPARG; PCK2
Wnt signaling pathway	0.00126	0.0999	APC2; CAMK2B; MMP7; FZD9; WNT9B; CACYBP; DKK1; DKK2; SFRP1; SFRP2; APC; TBL1XR1; BAMBI; RSPO3; GPC4; LGR5
Fatty acid metabolism	0.0018	0.102	ELOVL3; FASN; ACAT2; FADS2; HADHB; HSD17B4; SCD; FADS1

**Non-macular AMD RPE/choroid vs. non-macular control RPE/choroid**			

Choline metabolism in cancer	0.00445	0.363	WASF1; WAS; PLA2G4C; AKT2; PIK3R3; DGKH; MAPK10
Regulation of actin cytoskeleton	0.00495	0.363	WASF1; DIAPH2; WAS; TMSB4X; ITGA3; SPATA13; PIK3R3; ITGA6; ITGAE; ARHGEF7; FGD3
Osteoclast differentiation	0.00511	0.363	OSCAR; IFNAR1; TYROBP; AKT2; PIK3R3; TGFB2; MAPK10; LCK
Influenza A	0.00796	0.363	HLA-DRB5; DNAJB1; IFNAR1; XPO1; AKT2; IL18; PIK3R3; PYCARD; IFNA10
Hypertrophic cardiomyopathy (HCM)	0.00836	0.363	PRKAB2; ITGA3; ITGA6; TGFB2; CACNA1C; DAG1

Among the identified significant genes associated with the neuroactive ligand-receptor interaction, 13 genes were found down-regulated in macular AMD RPE/choroid including *ADRA1A, LEPR, PENK, SCT, BDKRB1, ADRB3, PTGDR, BDKRB2, RLN3, C5, EDN3, GABRE*, and *NPY1R*. *LEPR* or Leptin Receptor Factor was the second highest significant down-regulated gene. LEPR was initially identified as a satiety factor, but was subsequently shown to play a role in normal aging and neuroprotective processes (Gorska et al., 2010; Seshasai et al., 2015; Wauman et al., 2017). Other genes upregulated in the neuroactive ligand-receptor interaction pathway included *GRIK3, GRPR, CHRNA1, ADRA1D, OXTR, NPFFR1, P2RY2, MC5R, GABRB1, GRIA1, TRH, GCGR, MTNR1A, HTR2A, GRIN2C, CHRNB4*, and *GABRG3*.

All 12 significant genes associated with the ECM-receptor interaction pathway were upregulated in macular AMD RPE/choroid, with a distinct sub-pathway represented by a group of collagen genes including *COL6A3, COL9A3*, and *COL9A2.* The most statistically significant gene in the ECM group was *TNC* or Tenascin C, which encodes a key ECM component in the nervous system altered in various eye diseases (Kobayashi et al., 2016). Tenascin C also plays a role in inflammation process by regulating transforming growth factor β (TGFβ) (Reinhard et al., 2017). Noteworthy, *TGFB2* gene, an isoform of TGFβ, was also identified as up-regulated in both macular and non-macular meta-gene lists. Although not reaching statistical significance in this analysis, the fatty acid metabolism pathway was also among the enriched pathways in macular AMD RPE/choroid. Remarkably, all meta-genes associated with this pathway, consisting of *ELOVL3*, *FASN, ACAT2, FADS2, HADHB, HSD17B4, SCD*, and *FADS1*, were not differentially expressed in non-macular AMD RPE/choroid.

### PPI Network Analysis

**FIGURE 2:**
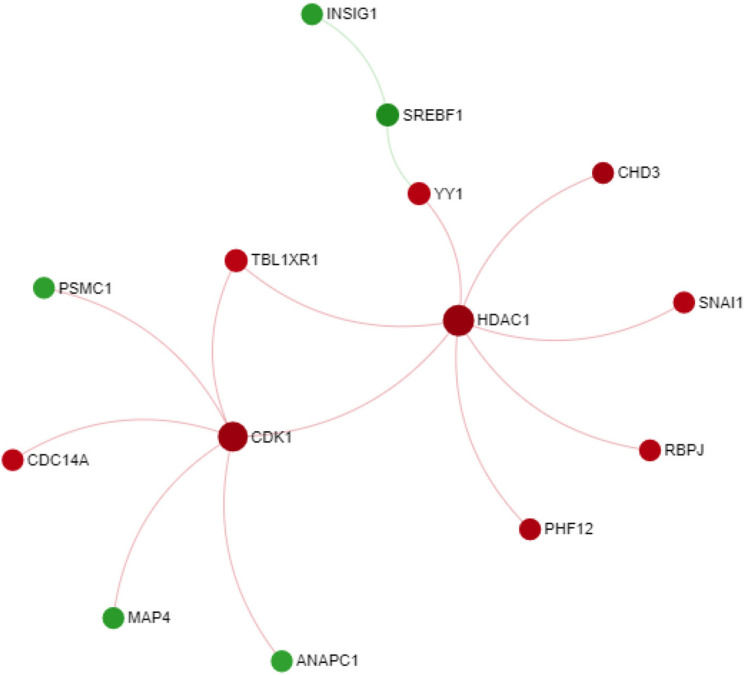
Zero order PPI network of meta-gene in macular AMD RPE/choroid. Downregulated nodes in red; upregulated nodes in green.

Since the macula is the primary anatomical area affected in AMD, we sought to get more insight into the genes differentially expressed in macular AMD RPE/choroid by further exploring them by through a PPI network. For this purpose, a PPI network was constructed using STRING database and Networkanalyst web-based tools, with the input of 764 significant genes from macular AMD RPE/choroid meta-gene list. Initially, a first order network created an extensive network comprising 1718 nodes and 2578 edges. To improve the clarity of the network and obtain more important nodes, we created a zero order PPI network (Figure 2). This network contains 14 nodes with the highest degree of 7. Two downregulated genes with the highest degrees and high centrality were Histone Deacetylase 1 (*HDAC1*) and Cyclin-dependent kinase 1 (*CDK1*). HDAC1 and CDK1 are both cell cycle regulators (Göder et al., 2018) suggesting altered cell proliferation responses in macular AMD RPE/choroid. We also input these 764 DE genes in AMD macular RPE/choroid into the WEB-based pathway analysis tool “GEne SeT AnaLysis Toolkit.” GEne SeT AnaLysis Toolkit constructs networks by using Network Topology-based Analysis method and used PPI BIOGRID as its reference list (Liao et al., 2019). The result revealed HDAC1 and CDK1 among the top five per cent of these genes when ranked by random walk probability (Supplementary Table S8).

## Discussion

The increasing microarray and RNAseq transcriptomic datasets available provide an important resource for exploring, at a molecular level, the pathogenic machinery of AMD through bioinformatics approaches (Morgan and DeAngelis, 2014; Tian et al., 2015). However, analysis of individual AMD transcriptomic datasets with conventional statistical approaches may not enable comprehensive identification of DE genes and pathways in functionally impaired RPE/choroid. For example, the microarray analysis undertaken by Whitmore et al. (2013) concluded that there were no significantly DE genes when FDR was applied to the respective AMD RPE/choroid dataset. Similarly, the RNAseq analysis described by Orozco et al. (2020) also highlighted less than 30 putative causal genes for AMD RPE/choroid. Analysis approaches combining different transcriptomic datasets obtained from different platforms were recently used to detect more DE genes in various diseases, such as dilated cardiomyopathy (Alimadadi et al., 2020), Alzheimer’s disease (Su et al., 2019), tuberculosis (Wang et al., 2018), rheumatoid arthritis (Badr and Häcker, 2019), and helminth infection (Zhou et al., 2016). These integrated analyses expand the number of specimens analyzed and are also well suited for AMD, given the multifactorial nature of the disease.

Here we report an analysis of normal and AMD RPE/choroid transcriptome data performed by integrating microarray and RNAseq datasets employing the web-based tool Networkanalyst (Xia et al., 2015) with Fischer’s method (Fisher, 1992; Alimadadi et al., 2020). Our analysis extended the number of statistically significant differentially expressed RPE/choroid genes in AMD to 764 in macular RPE/choroid, and 445 in non-macular AMD RPE/choroid. The resulting meta-genes identified as significantly differentially expressed in macular AMD RPE/choroid in comparison with normal RPE/choroid highlighted two significantly enriched pathways of potential functional importance in AMD pathogenesis, the neuroactive ligand-receptor interactions and extracellular matrix (ECM)-receptor interactions.

The most significant pathway in macular AMD RPE/choroid, the neuroactive ligand-receptor interactions had a FDR equal to 0.0297 by ORA analysis. This pathway regulates multiple neuroreceptors and their associated distant signaling molecules such as leptin, thyrotropin releasing hormone (TRH) and epinephrine (Biernacka et al., 2013; Kanehisa et al., 2016). It was previously shown to be functionally significant in neurotransmitter-mediated disorders such as alcohol dependence disorder (Biernacka et al., 2013), autism spectrum disorders (Wen et al., 2016), Parkinson’s disease (Hardy, 2010; Hamza et al., 2011; Kong et al., 2015), as well as some types of lung cancer (Ji et al., 2018). Our analysis suggested that 30 genes associated with this pathway may be linked to AMD, including *LEPR*, a receptor of leptin, which was initially identified in adipocytes (Gorska et al., 2010). Noteworthy, decreased serum leptin was observed in AMD patients in a case-control study and leptin was hypothesized to have a neuroprotective function and to lower the risk of AMD by removing extracellular β-amyloid in drusen deposits, decreasing triglyceride fatty acid synthesis and downregulating genes such as lipogenic enzyme, oxidative stress and inflammation related genes (Seshasai et al., 2015; Wauman et al., 2017). Our integrated data analysis identified the downregulation of leptin receptor in macular RPE/choroid in AMD for the first time. Cholinergic Receptor Nicotinic Alpha 1 Subunit (*CHRNA1*) and Cholinergic Receptor Nicotinic Beta 4 Subunit (*CHRNB4*), encoding two of the twelve gene subunits of the nicotinic acetylcholine receptor (Conti-Fine et al., 2000; Barrie et al., 2016), were found upregulated among the AMD meta-genes. The increased expression of these genes is associated with higher risk of lung cancer in smokers as the binding of the receptor by nicotine can stimulate angiogenesis especially within a context of inflammation and tumorigenesis (Yoo et al., 2014). The upregulation of *CHRNA1* and *CHRNB4* in AMD RPE/choroid may underlie one mechanism that contributes to the increased risk of AMD in smokers. Thyroid releasing hormone (TRH) has a central role in the thyroid hormone pathway that is found abnormal in some AMD patients. (Gopinath et al., 2016; Yang et al., 2018; Ma et al., 2020). Our analysis also showed that TRH, another gene linked to the neuroactive ligand receptor pathway, is upregulated in the AMD RPE/choroid.

Genes associated with the ECM-receptor interaction pathway in AMD, highlighted by our analysis, have previously been shown to have high variability of expression between individuals (Booij et al., 2009). The finding of multiple significantly upregulated genes associated with this pathway in AMD RPE/choroid underpins wound healing responses as putative pathophysiological mechanisms implicated in AMD (Newman et al., 2012). Tenascin C, the most statistically significant differentially expressed gene in this pathway, can upregulate TGFβ and promote inflammatory processes (Reinhard et al., 2017), in line with the increased level of Tenascin C identified in surgically excised choroidal neovascular membranes (Nicolò et al., 2000) and observation of its secretion in neovascular AMD (Kobayashi et al., 2016; Reinhard et al., 2017). Furthermore, although the fatty acid metabolism pathway was not found to be statistically significantly associated with AMD in our analysis, the finding that all differentially expressed genes in this pathway were found exclusively in macular RPE/choroid underlines the geographical differences in gene expression patterns between macular and non-macular RPE/choroid regions, previously suggested by Whitmore et al. (2014) and Ashikawa et al. (2017). Specific examples of genes with a macular pattern of differential expression were Fatty Acid Desaturase 1 (*FADS1*) and Fatty Acid Desaturase 2 (*FADS2*), genes encoding delta-5 and delta-6 desaturases, implicated in drusen formation in a recent study (Ashikawa et al., 2017). Hence, fatty acid metabolism abnormalities may contribute to drusen formation, an area of interest following the suggestion of secretion by the RPE of the lipid component of soft drusen, a hallmark of AMD progression (Curcio, 2018a,b).

The PPI network analysis highlighted two central hub genes involved in the control of cell proliferation/differentiation processes, *HDAC1* and *CDK1*. *HDAC1* encodes an isoform of histone deacetylase that is ubiquitously expressed and has a role in transcriptional repression (Hassig et al., 1998). Modification of chromatin structure through histone deacetylation has been identified as a mechanism of epigenetic regulation associated with various neurodegenerative diseases (Anderson et al., 2015). HDAC family members are involved in multiple biological processes including angiogenesis, inflammation and cell cycle progression, all of which play an important role in the pathophysiology of AMD (Tang et al., 2013). Noteworthy in this respect are the findings from a comparative study of Alzheimer’s disease and AMD donors that showed that *HDAC1, 2, 5*, and *6* expression decreased in the retina and frontal cortex of affected individuals (Noh et al., 2008). The other hub node identified, *CDK1* or cyclin-dependent kinase 1 plays an important role in the regulation of mitotic transition and phosphorylation of Bcl-2, Bcl-XL, and Mcl–1 proteins (Harley et al., 2010; Terrano et al., 2010). In the context of AMD, a retinal transcriptome analysis of senescence-accelerated OXYS rats revealed a possible role of CDK1 in the retinal extrinsic apoptotic processes associated with AMD. Specifically, the study associated the increased apoptotic activity with *CDK1*, which was identified as a hub gene for functional clusters associated with the MAPK and p53 signaling pathways in the interaction network constructed from the respective transcriptomic data (Telegina et al., 2015).

A limitation of this analysis is due to the paucity of samples representing the individual disease stage phenotypes and respective subgroup analyses of AMD (early, intermediate, advanced) in the original studies (Supplementary Figure S3) resulting in reduced power and the ensuing application of pathway analyses on combined datasets of mixed disease stages. Thus the advanced AMD refers here to mixed advanced stages of AMD (both GA and NV AMD). Clearly, an increase in the clinical data available with post-mortem RPE/choroid samples used in omic technologies could enable more detailed studies into the pathophysiological processes particular to each stages of AMD highlighting key progression factors to target for further therapeutic intervention research (Handa et al., 2019).

In conclusion, integration of microarray data and RNAseq data allows transcriptomic analyses of increased power and identification of DE meta-genes in AMD RPE/choroid. Taking such an approach, this study identified two novel pathways characterized by significant enrichment of DE genes in AMD RPE/choroid, namely the neuroactive-ligand receptor interaction pathway and the ECM-receptor interaction pathway. In addition, the PPI network analysis highlighted two hub nodes that may link apoptotic and angiogenesis pathological processes in AMD. The integrated functional analysis of DE genes in AMD also revealed genes previously linked to other neurodegenerative disease such as Alzheimer’s disease and Parkinson’s disease. The approach used to integrate publicly available transcriptomic datasets obtained through different experimental platforms provided a novel insight and broadened the exploration of a larger number of potential genes and functional pathways with roles in AMD pathogenesis.

## Data Availability Statement

Publicly available datasets were analyzed in this study. This data can be found here: “Newman, A. M., Gallo, N. B., Hancox, L. S., Miller, N. J., Radeke, C. M., Maloney, M. A., et al. (2012). Systems-level analysis of age-related macular degeneration reveals global biomarkers and phenotype-specific functional networks. Gene Expression Omnibus. GSE29801 and Orozco, L. D., Chen, H. H., Cox, C., Katschke, K. J., Arceo, R., et al. (2020). Integration of eQTL and a Single-Cell Atlas in the Human Eye Identifies Causal Genes for Age-Related Macular Degeneration. Gene Expression Omnibus. GSE135092.”

## Author Contributions

DD, XL, and LP designed the study and wrote the manuscript. DD and XL performed the data analysis. All authors discussed the results, reviewed and approved the final version of manuscript.

## Conflict of Interest

The authors declare that the research was conducted in the absence of any commercial or financial relationships that could be construed as a potential conflict of interest.

## Footnotes

1 https://www.networkanalyst.ca/

## Supplementary Material

FIGURE S1 PCA plot of macular AMD RPE/choroid vs. control RPE/choroid representing the datasets before and after applying ComBat algorithm.

FIGURE S2 PCA plot of non-macular AMD RPE/choroid vs. control RPE/choroid representing the datasets before and after applying ComBat algorithm.

FIGURE S3 Venn diagram showing the overlap of differentially expressed genes identified by subgroup analysis in macular locations of RPE/choroid in AMD.

TABLE S1 Transcriptome studies of postmortem human AMD RPE/choroid.

TABLE S2 Comparison of differential expressed genes in macular AMD RPE/choroid.

TABLE S3 Comparison of differential expressed genes in non-macular AMD RPE/choroid.

TABLE S4 Meta-gene list of differential expressed genes in macular AMD RPE/choroid.

TABLE S5 Meta-gene list of differential expressed genes in non-macular AMD RPE/choroid.

TABLE S6 Similar meta-genes between macular and non-macular AMD RPE/choroid.

TABLE S7 Comparison of differential expressed genes in different subgroups of macular AMD RPE/choroid.

TABLE S8 Meta-gene in macular AMD RPE/choroid ranking by random walk probability using Network Topology-based Analysis.
