# Supplementary figures and images for: Integrated Microarray and RNAseq Transcriptomic Analysis of Retinal Pigment Epithelium/Choroid in Age-Related Macular Degeneration
Source: Front Cell Dev Biol. 2020 Aug 21;8:808. doi: 10.3389/fcell.2020.00808 (PMC7480186; doi:10.3389/fcell.2020.00808)

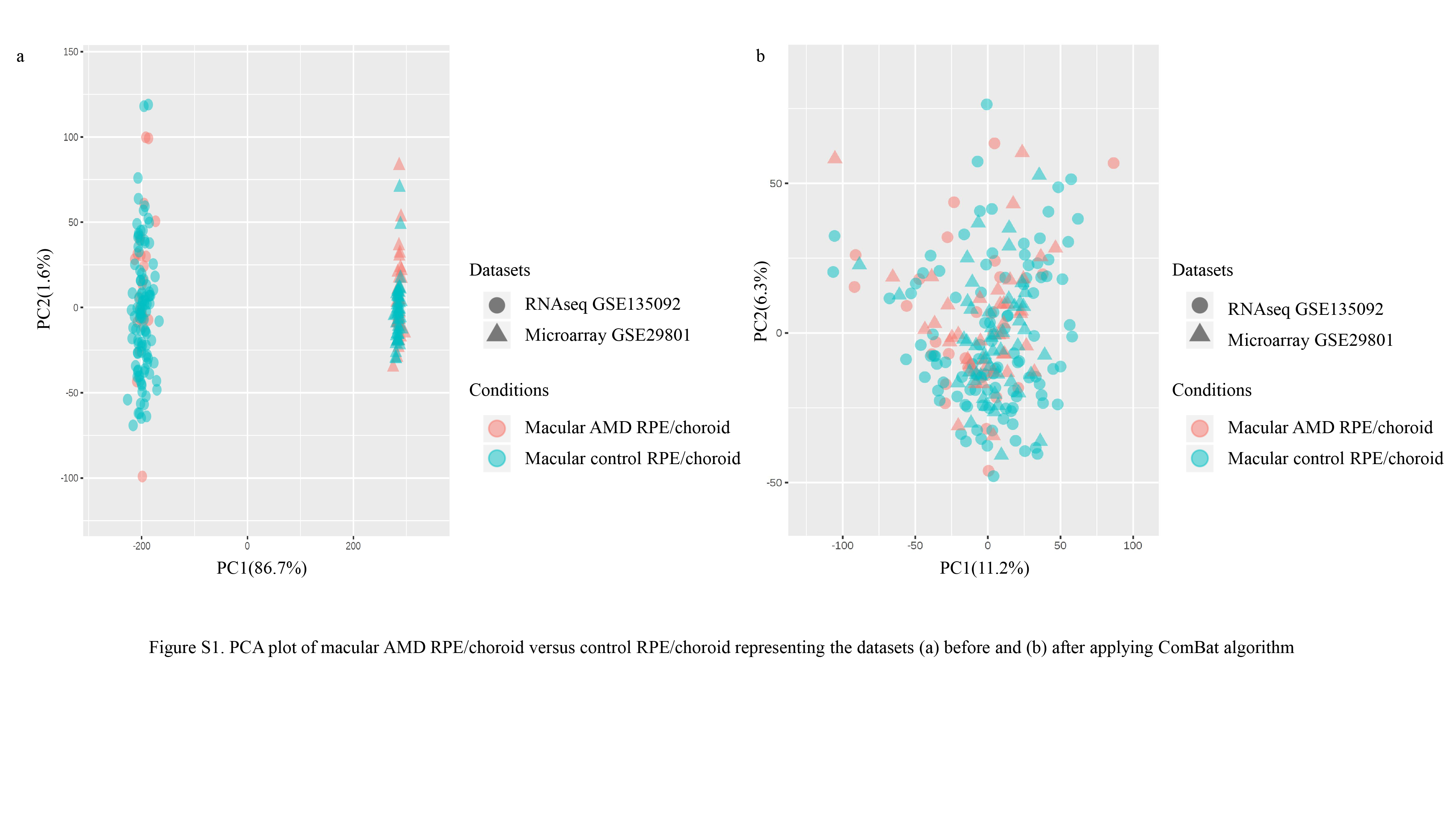

Supplement: FIGURE S1 — PCA plot of macular AMD RPE/choroid vs. control RPE/choroid representing the datasets before and after applying ComBat algorithm. [file Image_1.JPEG]

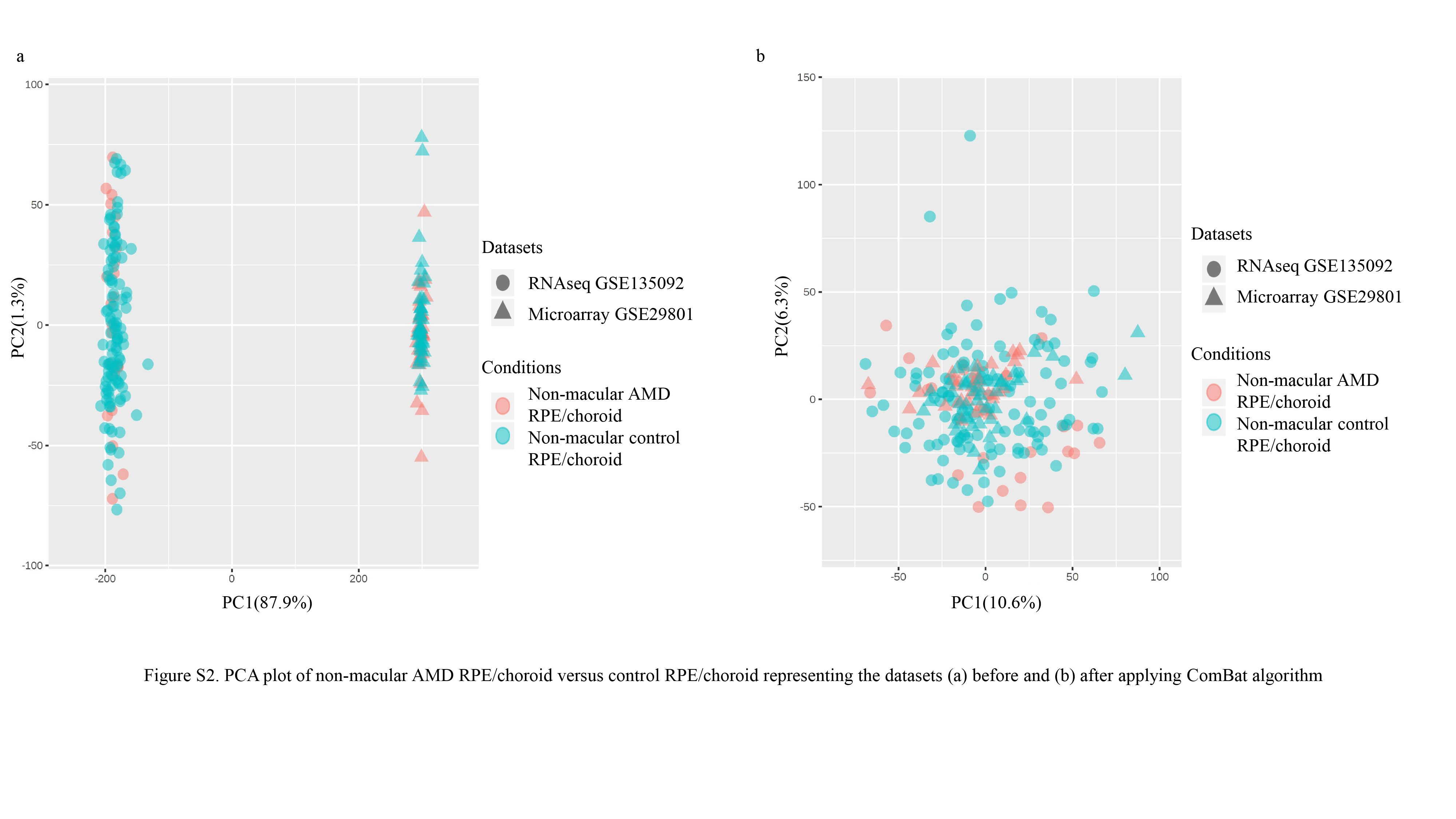

Supplement: FIGURE S2 — PCA plot of non-macular AMD RPE/choroid vs. control RPE/choroid representing the datasets before and after applying ComBat algorithm. [file Image_2.JPEG]

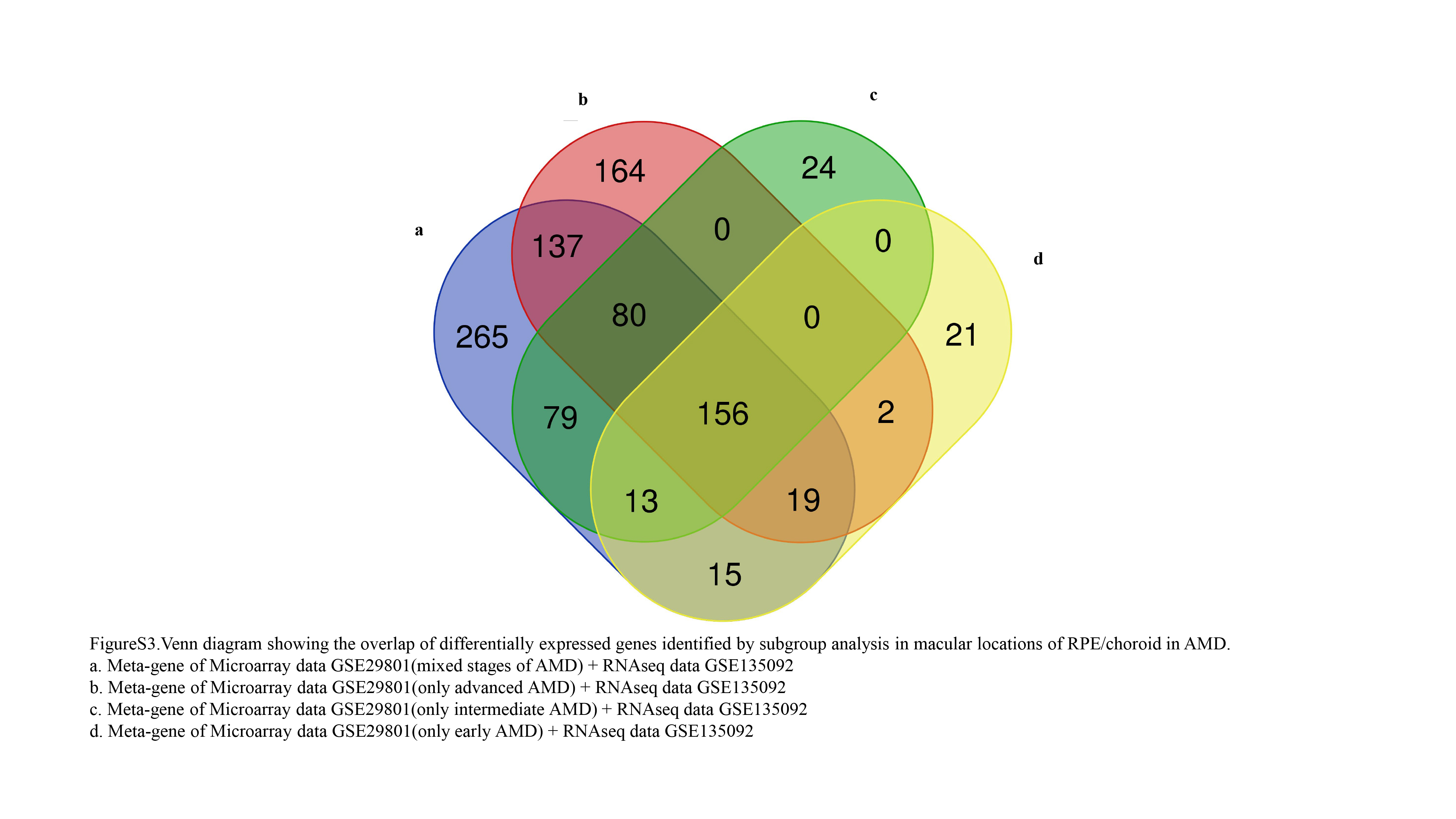

Supplement: FIGURE S3 — Venn diagram showing the overlap of differentially expressed genes identified by subgroup analysis in macular locations of RPE/choroid in AMD. [file Image_3.JPEG]
